# Safety of intratumoral immunostimulatory LOAd703 gene therapy combined with chemotherapy in patients with advanced cancer

**DOI:** 10.1016/j.iotech.2026.101585

**Published:** 2026-02-06

**Authors:** A. Hahn, S. Irenaeus, L.C. Sandin, J. Wenthe, E. Eriksson, J.L. Jarblad, A. Schiza, H. Dahlstrand, U. Olsson-Strömberg, J. Krause, A. Sundin, A. Loskog, G.J. Ullenhag

**Affiliations:** 1 Department of Immunology, Genetics and Pathology, Uppsala University, Uppsala, Sweden; 2 Department of Oncology, Uppsala University Hospital, Uppsala, Sweden; 3 Lokon Pharma AB, Uppsala, Sweden; 4 Department of Oncology-Pathology, Karolinska Institute, Stockholm, Sweden; 5 Department of Medical Sciences, Uppsala University Hospital, Uppsala, Sweden; 6 Department of Hematology, Uppsala University Hospital, Uppsala, Sweden; 7 Department of Surgical Sciences, Radiology & Molecular Imaging, Uppsala University, Uppsala, Sweden

**Keywords:** gene therapy, tumor microenvironment, oncolytic virus, CD40L, 4-1BBL

## Abstract

**Background:**

LOAd703 is a tumor microenvironment (TME) gene-engineering adenovirus encoding the immunostimulatory transgenes trimerized membrane-bound CD40 ligand (CD40L) and 4-1BB ligand (4-1BBL). Upon administration in the TME, the transgenes are expressed in various cell types to engage both the tumor and its stroma to activate antitumor immunity. CD40–CD40L interaction causes dendritic cell maturation and stimulates T helper 1-type immune responses, whereas 4-1BB–4-1BBL signaling protects T cells and natural killer cells from activation-induced cell death and promotes lymphocyte proliferation. LOAd703 replication with subsequent oncolysis is restricted to cancer cells.

**Patients and methods:**

In the dose-escalating part of this clinical study (NCT03225989), LOAd703 was increased according to a standard 3 + 3 design in patients with advanced solid malignancies. LOAd703 was administered every 2 weeks by ultrasound-guided intratumoral injections, combined with a standard-of-care or immune-conditioning gemcitabine-based chemotherapy regimen. The primary endpoint was tolerability.

**Results:**

Three dose levels of LOAd703 were evaluated in 10 patients. Treatment was overall safe and well tolerated. The most common side-effects assessed as secondary to LOAd703 were pyrexia, fatigue and headache. All LOAd703-attributed adverse events were of grade 1-2, and the majority were transient and emerged shortly after administration. One patient developed cytokine release syndrome grade 2. The maximum tolerated dose was not reached. Median overall survival was 8.4 months, and the overall response rate was 20%. A trend of higher interferon-gamma (IFN-γ) plasma levels in the highest LOAd703 dose cohort was observed.

**Conclusion:**

The acceptable toxicity associated with LOAd703 and chemotherapy, combined with signs of clinical benefit in poor prognostic cancer patients, warrant further studies.

## Highlights

• LOAd703 demonstrated a tolerable safety profile in advanced colorectal, pancreatic and ovarian cancer.
• LOAd703-related adverse events were all of grade 1-2, most were transient and emerged adjacent to the day of administration.
• Two pancreatic cancer patients experienced partial response, and one ovarian cancer patient had stable disease ≥6 months.
• The highest LOAd703 dose cohort showed a trend of higher IFN-γ plasma levels after LOAd703 treatment.

## Introduction

Immunotherapy in the form of checkpoint blockade antibodies, which releases the brakes on pre-existing tumor immunity, has become one of the cornerstones in cancer treatment alongside surgery, chemotherapy and radiotherapy. However, primary and secondary resistance is common and there are multiple poor prognostic cancer types which do not respond to checkpoint blockade.^1^ Various therapeutic targets in the tumor microenvironment (TME) are being investigated with the aim to enhance immune activation as a monotherapy or to sensitize the tumor to combination checkpoint blockade.^2^ The currently available immunotherapies are almost exclusively administered systemically. However, for *in vivo de novo* immune-activating therapies, there are several potential advantages in delivering immunotherapy locally with the aim of achieving a systemic antitumor effect. Intratumoral administration of an immune activator allows a high concentration of the immunostimulatory drug locally in the TME while the systemic exposure to the drug remains low, which reduces the risk of systemic side-effects. Moreover, this approach utilizes the injected tumor’s antigens to drive immunity and potentially elicits a broad antitumor immune response capable of targeting both the injected and non-injected lesions.^3^

Intratumoral administration of an immunostimulatory gene therapy enables the transfer of genes encoding immunostimulatory proteins into the injected tumor. CD40 ligand (CD40L; CD154) and 4-1BB ligand (4-1BBL; CD137L) are both immunostimulatory proteins that drive T helper 1 (Th1)-type immune responses and represent interesting candidates for cancer immunotherapy. CD40L is found on many different cell types and the binding to its receptor CD40 has pleiotropic effects depending on the identity of the CD40+ cell. The binding of CD40L to its receptor CD40 on immature dendritic cells (DCs) transforms them into highly effective antigen presenting cells with the capacity to stimulate Th1, cytotoxic T lymphocytes, natural killer (NK) cells and pro-inflammatory macrophages. CD40L binding to CD40+ cancer cells, on the other hand, causes growth inhibition or apoptosis.^4^ 4-1BBL binds to its receptor 4-1BB (CD137) which is found on activated T and NK cells. Signaling through this receptor–ligand pair protects T and NK cells from activation-induced cell death by up-regulation of apoptosis inhibitors such as B-cell lymphoma-extra large and promotes proliferation, cytokine production and activation of memory T cells.^5^

LOAd703 (delolimogene mupadenorepvec) is a novel immunotherapy for cancer. It is a TME gene-engineering vector based on a replication-competent adenovirus serotype 5/35 encoding both a trimerized, membrane-bound form of CD40L (TMZ-CD40L) and 4-1BBL under the control of a cytomegalovirus (CMV) promoter. Upon intratumoral delivery, TMZ–CD40L and 4-1BBL will be expressed by various cells in the TME due to the promiscuous CMV promoter. Hence, all infected cells will provide immune-activating signals to nearby DCs, T and NK cells in the TME.^6^ A murine version of LOAd703 has further been shown to induce infiltration of CD103+ DCs, CD8+ T cells and NK cells when administered intratumorally in immunocompetent mice.^7^ LOAd703 induces the expression of CCR7 on DCs which enables their migration to lymph nodes and subsequent systemic immune activation.^8^^,^^9^ Further, the infected tumor cells can release exosomes that carry both TMZ-CD40L and 4-1BBL in protein form and as messenger RNA (mRNA). Commonly, exosomes are also loaded with tumor antigens. These exosomes can travel and have the capacity to stimulate DCs that they encounter which further promotes activation of systemic immunity.^10^ LOAd703 has been modified to restrict virus replication and subsequent oncolysis to cells with a dysfunctional retinoblastoma pathway, i.e. the tumor cells only. Studies with LOAd703 have demonstrated its capacity to induce oncolysis in cell lines derived from various solid malignancies while sparing healthy tissue.^6^^,^^11^ The DC-mediated uptake of dying tumor cells together with the immune stimulation provided by the transgenes create a personalized vaccine for each individual patient treated.

Chemotherapies may enhance the effectiveness of immunotherapy. Gemcitabine is one such example as it has been shown to reduce immunosuppressive cell populations and decrease transforming growth factor-beta (TGF-β) in peripheral blood while maintaining effector T-cell proliferation.^12^^,^^13^ Patients in our study were treated with LOAd703 combined with either immune-conditioning gemcitabine or a standard-of-care gemcitabine-based regimen. Gemcitabine was considered immune-conditioning when it was given only because of its immunomodulating features and not expected to induce effective direct antitumor responses such as when refractory to previous gemcitabine treatment or when not included as a standard-of-care option. Due to the small heterogeneous population in this study, and lack of a control group, it is only possible to determine the safety of LOAd703 combined with chemotherapy and not whether chemotherapy conditioning potentiates the effect of LOAd703.

LOAd703 has been tested in three different trials in the United States and Sweden (NCT02705196, NCT03225989 and NCT04123470). A study with locally advanced or metastatic pancreatic cancer is the only reported so far. It demonstrated that LOAd703 combined with gemcitabine and nab-paclitaxel was feasible, and the most common adverse events (AEs) were pyrexia, fatigue, chills and elevated liver enzymes. The overall response rate was 38% in the intention-to-treat population.^14^ The current report presents safety and efficacy signals (immunological and clinical) of dose-escalating LOAd703 in patients with advanced colorectal, pancreatic and ovarian cancer in a clinical study. LOAd703 was administered by repeated intratumoral injections together with either standard-of-care or immune-conditioning chemotherapy.

## Patients and methods

### Study design

LOKON002 is an open-label, single-arm, phase I/II study evaluating increasing doses of intratumorally injected LOAd703 combined with chemotherapy. It was conducted in accordance with the principles for the Declaration of Helsinki and the International Conference on Harmonization guidelines for Good Clinical Practice. The study was approved by the Swedish Ethical Review Authority (approved 2017-12-13, reference number 2017/327). Patients provided written informed consent before enrollment. The first 10 patients were subjected to dose escalation, and data from this cohort are presented in this report with the following primary endpoint: tolerability using Common Terminology Criteria for Adverse Events (CTCAE) version 4.03, and the following secondary endpoints: local and distant antitumoral effects according to RECIST 1.1 and overall survival (OS). A full study description can be found at www.clinicaltrials.gov (NCT03225989).

Eligible patients were patients with colorectal, pancreatic, biliary or epithelial ovarian cancer (including epithelial ovarian, fallopian tube or primary peritoneal carcinoma). All patients, irrespective of primary tumor, had to be aged ≥18 years, have a 0-2 Eastern Cooperative Oncology Group performance status, have a disease burden roughly defined as a total tumor burden of ≤70 cm^2^, have measurable disease per RECIST 1.1 criteria and have at least one non-irradiated lesion (or irradiated but disease progression documented at the site after radiation) suitable for image-guided intratumoral injection and needle biopsy. Moreover, all patients had to have no remaining acute toxic effects from previous anticancer treatment >grade 1 and have adequate baseline organ and hematological function. Concurrent treatment that could compromise the study including high-dose corticosteroids (>10 mg/24 h of prednisolone or equivalent) and active, severe autoimmune disease were among the exclusion criteria.

This phase I study had a standard 3 + 3 design in which a maximum of eight treatments with LOAd703 were administered by ultrasound-guided intratumoral injections every 2 weeks. The prescribed LOAd703 dose was injected into one predetermined lesion per cycle in all patients even if it was allowed to select up to three lesions. It was allowed to change lesion for injection if the initial one for some reason became unsuitable for injection, e.g. size reduction. Lesions were chosen based on accessibility for injection and biopsy without injuring the intestines or large vessels, as well as being measurable by radiology. Pancreatic cancer patients could receive LOAd703 combined with standard-of-care gemcitabine (1000 mg/m^2^) and nab-paclitaxel (125 mg/m^2^) on days 1, 8 and 15 in 28-day cycles until progressive disease (PD) or unacceptable toxicity. Patients with the remaining cancer types, including pancreatic cancer patients in whom standard-of-care chemotherapy was not applicable, received LOAd703 combined with immune-conditioning gemcitabine. Conditioning gemcitabine (1000 mg/m^2^) was given on day 1, 8 and 15 in 28-day cycles and was stopped after the final virus dose. Chemotherapy, either standard-of-care gemcitabine plus nab-paclitaxel or conditioning gemcitabine, was administered after virus injection when given the same week, and preferably initiated within 6 h after LOAd703 injection. Dose reductions or omissions were allowed. The planned LOAd703 dose escalation in phase I was: 5 × 10^10^, 1 × 10^11^ and 5 × 10^11^ viral particles (VP).

### Assessments

Symptoms at baseline were registered, and AEs were recorded from baseline until last study visit (up to 40 weeks). AEs were coded utilizing the Medical Dictionary for Drug Regulatory Activities (MedDRA) and graded from 1 to 5 according to the National Cancer Institute’s CTCAE version 4.03. Each AE was designated as unrelated, unlikely, possibly, probably or definitely attributed to potential causes such as LOAd703, injection procedures or chemotherapy. The probability level of an AE being attributed to the chemotherapy regimen versus LOAd703 was based on previous knowledge of the chemotherapies as well as other immunotherapies, including studies of immunotherapies with a similar mechanism of action. Each dose cohort had to comprise at least three patients who were assessable for a dose-limiting toxicity (DLT). DLT was defined according to CTCAE version 4.03 as an AE grade 3 or higher that could be attributed (definitely, probably or possibly) to LOAd703, either as the only cause or a contributing one. As the chemotherapy regimens used in this study are already approved, they were not part of the DLT evaluation. The DLT period lasted from the first injection of LOAd703 until 2 weeks after the third injection. Possible contribution of LOAd703 to a DLT precluded continuation of treatment with the investigational product in that patient unless the occurring DLT was transient fever grade 3, nausea, vomiting or increased liver enzymes, in which case dose reduction according to study protocol could be considered.

Radiological evaluation according to RECIST 1.1 was carried out every 2 months as per clinical routine for up to 40 weeks from the first LOAd703 injection. OS was followed for all patients until data cut-off (27 May 2025). Blood was sampled at baseline, week 7 and week 13 to evaluate immune biomarkers. Tumor tissue was sampled before LOAd703 injection at baseline and at week 13, and the results will be presented in a separate paper with biomarker analyses.

### LOAd703

LOAd703 was manufactured at Baylor College of Medicine, Houston, Texas, and stored at −70°C. The viral suspension (Tris/glycerol buffer, pH 8) was thawed and kept at 4°C for a maximum of 24 h before injection. Sterile physiological saline (sodium chloride 0.9%) was used if dilution of the viral suspension was necessary. For injections, a 1-ml syringe with a Luer lock and a 20G, 120-mm needle were prepared with the virus. The final injected volume was 0.5 ml.

### MesoScale

Plasma samples were analyzed for cytokine and chemokine expression with a V-Plex chemokine panel 1 and a customized U-Plex panel [chemokine C-X-C motif ligand 1, interferon-gamma (IFN-γ), interleukin (IL)-1β, IL-6, IL-8, IL-10, IL-12p70, IL-15, tumor necrosis factor-α, vascular endothelial growth factor-α] from MesoScale Diagnostics (Rockville, MD). The assays were carried out according to the manufacturer’s protocol.

### Statistical evaluations

No statistical calculations were done for clinical data as they were only descriptive at this stage. Cytokine expression measured with MesoScale was analyzed with two-way analysis of variance (ANOVA) comparing dose levels at each time point and one-way ANOVA comparing time points across dose levels, both using *post hoc* Tukey’s multiple comparison test.

## Results

### Patient characteristics

Ten patients were enrolled at Uppsala University Hospital between April 2018 and November 2019 and comprised seven men and three women with a median age of 54 years (range 33-73 years). The patients had colorectal cancer (*n* = 4), pancreatic cancer (*n* = 4) and ovarian cancer (*n* = 2). The majority were heavily pretreated before inclusion, with a median of 2 (range 0-5) treatment lines. Baseline characteristics are shown in Table 1. Three patients (PC-8-10) with pancreatic cancer received intratumoral injections of LOAd703 combined with standard-of-care treatment (gemcitabine ± nab-paclitaxel). The remaining patients received LOAd703 in combination with immune-conditioning gemcitabine. As the injected volume with LOAd703 was small (0.5 ml), the full dose was delivered to all patients at all treatment occasions. Only one lesion was injected per treatment occasion. The vast majority of patients (*n* = 9) had metastatic disease at baseline. Eight patients had liver metastases, and seven of them received intratumoral injections into the liver, of which one also got injected into a tumor-engaged lymph node. One patient received injections in a lymph node only and two patients received injections in the primary tumor of the pancreas. Commonly the same lesion was injected, but if it disappeared or otherwise became difficult to reach, a new lesion could be selected. The lesion used for injections was changed in two patients, CRC-4 and OC-7. In CRC-4 the first lesion became difficult to identify and in OC-7 the first lesion became necrotic. The remaining patients received all injections in the same lesion. Each patient received between three and eight intratumoral injections. The reasons for patients (*n* = 5) not receiving all scheduled LOAd703 treatments were tumor progression and/or clinical deterioration. Treatment characteristics are shown in Table 2.

**Table 1:** Patient characteristics

Patient ID	Age	Sex	ECOG performance status	Primary tumor	Stage of disease	Time from diagnosis of advanced disease (months)	Prior systemic treatment lines for advanced disease (*n*)	LDH at TI (reference range^a^)
**Dose level: 5 × 10^10^ VP**								
PC-1	58	Male	0	Pancreatic	Metastatic	5	1	2.6 (1.8-3.4)
CRC-2	67	Male	1	Colorectal	Metastatic	56	3	5.1 (1.8-3.4)
CRC-3	42	Male	0	Colorectal	Metastatic	59	2	5.7 (1.8-3.4)
**Dose level: 1 × 10^11^ VP**								
CRC-4	53	Male	0	Colorectal	Metastatic	26	3	4.3 (1.8-3.4)
OC-5	33	Female	0	Ovarian	Metastatic	94	4	2.9 (1.8-3.4)
CRC-6	73	Female	1	Colorectal	Metastatic	36	4	2.0 (1.9-4.2)
**Dose level: 5 × 10^11^ VP**								
OC-7	49	Female	1	Ovarian	Metastatic	24	5	3.8 (1.8-3.4)
PC-8	55	Male	0	Pancreatic	Metastatic	10	2	2.7 (1.8-3.4)
PC-9	68	Male	0	Pancreatic	Locally advanced	0	0	2.4 (1.8-3.4)
PC-10	53	Male	0	Pancreatic	Metastatic	2	0	2.9 (1.8-3.4)

Individual characteristics for the 10 patients in LOKON002 subjected to dose escalation at enrollment are presented.

ECOG, Eastern Cooperative Oncology Group; LDH, lactate dehydrogenase; TI, treatment initiation; VP, viral particles.

a Reference range of LDH correlated to age.

**Table 2:** Treatment characteristics

Patient ID	LOAd703 injection site	Number of LOAd703 injections	Type of concomitant chemotherapy	Chemotherapy intention
**Dose level: 5 × 10^10^ VP**				
PC-1	Liver	8	Gemcitabine	Conditioning
CRC-2	Liver	5	Gemcitabine	Conditioning
CRC-3	Liver	4	Gemcitabine	Conditioning
**Dose level: 1 × 10^11^ VP**				
CRC-4	Liver	8	Gemcitabine	Conditioning
OC-5	Lymph node	8	Gemcitabine	Conditioning
CRC-6	Liver	8	Gemcitabine	Conditioning
**Dose level: 5 × 10^11^ VP**				
OC-7	Lymph node and liver	4	Gemcitabine	Conditioning
PC-8	Liver	3	Gemcitabine	Standard of care
PC-9	Pancreas	7	Gemcitabine + nab-paclitaxel	Standard of care
PC-10	Pancreas	8	Gemcitabine + nab-paclitaxel	Standard of care

Individual treatment data for the 10 patients in LOKON002 subjected to dose escalation are presented.

VP, viral particles.

### Safety of LOAd703

Common AEs assessed as definitely, probably or possibly related to LOAd703 were pyrexia, fatigue and headache (Table 3). AEs were commonly of low grade (grade 1-2) and transient. No AEs of high grade (grade 3-4) were assessed as being associated with LOAd703 treatment. Many of the reported AEs were assessed as related to disease or chemotherapy. All listed AEs are shown in Supplementary Table S1, available at https://doi.org/10.1016/j.iotech.2026.101585. Five serious adverse events (SAEs) in three patients graded 1-2 were related to LOAd703—four pyrexia events and one cytokine release syndrome (CRS). Each of these patients belonged to different dose cohorts, two of them had pancreatic cancer (PC-1 and PC-10) and one colorectal cancer (CRC-4). Furthermore, there was no obvious difference in AE frequency correlated to dose level (Table 3). Most AEs attributed to LOAd703 developed shortly after injection within a few hours up to 3 days, and only a few developed after 1-2 weeks. The AEs attributed to LOAd703 which developed the same day or the day after injection are shown per patient and number of injection in Supplementary Table S2, available at https://doi.org/10.1016/j.iotech.2026.101585.

**Table 3:** Adverse events related to LOAd703

Type of AE	Total, *N* (%) of patients^a^ with					Total number of AEs
	AE Grade 1-2	AE Grade 3-4	SAE Grade 1-2	SAE Grade 3-4	AE Any grade	
**Dose level: 5 × 10^10^ VP (*n* = 3)**						
Chills	3 (100)	0 (0)	0 (0)	0 (0)	3 (100)	7
Pyrexia	3 (100)	0 (0)	1 (33)	0 (0)	3 (100)	11
Fatigue	2 (67)	0 (0)	0 (0)	0 (0)	2 (67)	3
Myalgia	2 (67)	0 (0)	0 (0)	0 (0)	2 (67)	3
Lung infiltration	1 (33)	0 (0)	0 (0)	0 (0)	1 (33)	1
Malaise	1 (33)	0 (0)	0 (0)	0 (0)	1 (33)	1
Vomiting	1 (33)	0 (0)	0 (0)	0 (0)	1 (33)	3
**Dose level: 1 × 10^11^ VP (*n* = 3)**						
Fatigue	2 (67)	0 (0)	0 (0)	0 (0)	2 (67)	3
Headache	2 (67)	0 (0)	0 (0)	0 (0)	2 (67)	7
Pyrexia	2 (67)	0 (0)	0 (0)	0 (0)	2 (67)	2
C-reactive protein increased	1 (33)	0 (0)	0 (0)	0 (0)	1 (33)	1
Cytokine release syndrome	1 (33)	0 (0)	1 (33)	0 (0)	1 (33)	1
Discomfort	1 (33)	0 (0)	0 (0)	0 (0)	1 (33)	2
Hypotension	1 (33)	0 (0)	0 (0)	0 (0)	1 (33)	1
Lung infiltration	1 (33)	0 (0)	0 (0)	0 (0)	1 (33)	1
Myalgia	1 (33)	0 (0)	0 (0)	0 (0)	1 (33)	1
Nausea	1 (33)	0 (0)	0 (0)	0 (0)	1 (33)	1
Sinus tachycardia	1 (33)	0 (0)	0 (0)	0 (0)	1 (33)	1
Vomiting	1 (33)	0 (0)	0 (0)	0 (0)	1 (33)	1
**Dose level: 5 × 10^11^ VP (*n* = 4)**						
Pyrexia	4 (100)	0 (0)	1 (25)	0 (0)	4 (100)	14
Fatigue	3 (75)	0 (0)	0 (0)	0 (0)	3 (75)	4
Headache	2 (50)	0 (0)	0 (0)	0 (0)	2 (50)	3
Back pain	1 (25)	0 (0)	0 (0)	0 (0)	1 (25)	1
Blood creatinine increased	1 (25)	0 (0)	0 (0)	0 (0)	1 (25)	1
C-reactive protein increased	1 (25)	0 (0)	0 (0)	0 (0)	1 (25)	1
Chills	1 (25)	0 (0)	0 (0)	0 (0)	1 (25)	2
Diarrhea	1 (25)	0 (0)	0 (0)	0 (0)	1 (25)	1
Generalized edema	1 (25)	0 (0)	0 (0)	0 (0)	1 (25)	1
Hypotension	1 (25)	0 (0)	0 (0)	0 (0)	1 (25)	2
Injection site inflammation	1 (25)	0 (0)	0 (0)	0 (0)	1 (25)	1
Myalgia	1 (25)	0 (0)	0 (0)	0 (0)	1 (25)	1
Vomiting	1 (25)	0 (0)	0 (0)	0 (0)	1 (25)	1

AEs were graded according to CTCAE version 4.03. Data are *n* (%) unless otherwise specified. Worst grade is included in the table. Related AEs events are those reported as ‘possibly related’, ‘probably related’ or ‘definitely related’ to LOAd703. Coded according to the Medical Dictionary for Regulatory Activities (MedDRA v21.0).

AE, adverse event; CTCAE, Common Terminology Criteria for Adverse Events; SAE, serious adverse event; VP, viral particles.

a Safety-assessable patients. Data cut-off: 30 November 2023.

One event of CRS was registered. Patient CRC-4 developed SAE CRS grade 2. This patient had colorectal cancer and was treated with LOAd703 1 × 10^11^ VP which was injected in a liver metastasis. About 7 h after the third intratumoral injection with LOAd703, this patient experienced hypotension, with a lowest blood pressure of 75/45 mmHg, tachycardia with a pulse of 120 and fever of 38.1°C. The patient received 2.5 l of crystalloid intravenous fluids before tocilizumab. Symptoms of CRS subsided 30 min after tocilizumab was administered. Serum sampled 6 h after the development of CRS showed IL-6 levels of 40 ng/l and increased C-reactive protein (CRP) from 5 mg/l before study treatment to 22 mg/l (CRP reference <5 mg/l). There was also a tendency of increased creatinine from 69 μmol/l before study treatment to 84 μmol/l after study treatment and CRS management therapy (creatinine reference 60-105 μmol/l), indicating treatment-induced renal dysfunction. Serum sampled 2 days after the CRS showed IL-6 levels of 172 ng/l. Subsequent treatment with LOAd703 was given without complications after premedication with betamethasone. No isolated cases of hepatotoxicity, i.e. not part of CRS, were associated with LOAd703. Patient OC-05 developed two events of increased liver transaminases related to gemcitabine. The transaminase levels declined after gemcitabine was discontinued and normalized during treatment with LOAd703 alone.

No DLTs were reported and subsequently the maximum tolerated dose was not reached.

### Clinical results

**Figure 1:**
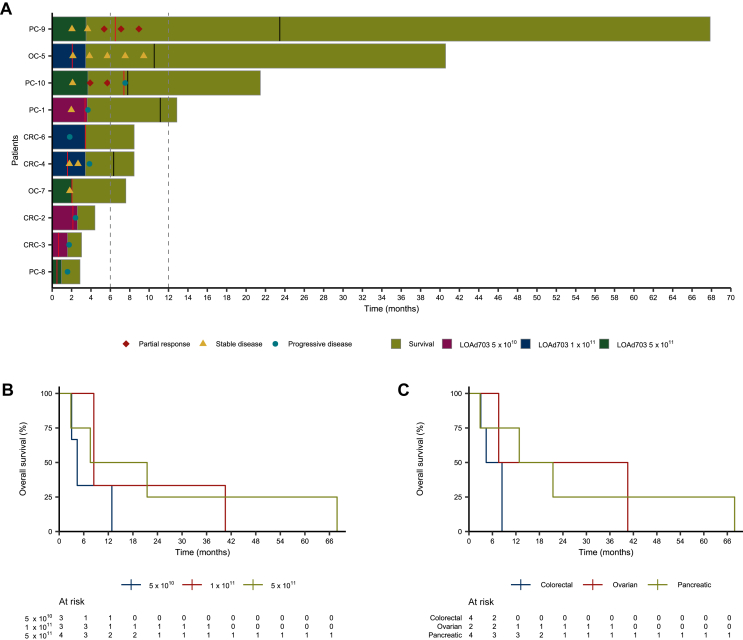
**Treatment and efficacy data during dose escalation of LOAd703 in LOKON002.** For overall survival, all patients (*n* = 10) reached the event during the observation period resulting in no censored observations. Survival was followed until data cut-off on 27 May 2025. (A) Swimmer plot which shows treatment characteristics and survival for individual patients. Patients were treated with a maximum of eight intratumoral injections with LOAd703 combined with conditioning or standard-of-care chemotherapy. Patients treated with LOAd703 combined with standard-of-care chemotherapy could continue to receive chemotherapy within the study after the last LOAd703 injection. Last dose of chemotherapy given within the study is marked with a red line. Start of new treatment, including study treatment, is marked with a black line. Efficacy measurements include overall survival and radiological response according to RECIST 1.1. Seventy percent of the patients survived ≥6 months, and 40% ≥12 months. PR was achieved in 20% of the patients. (B) Kaplan–Meier curve with overall survival by dose level. No clear difference could be detected between the dose-level groups in this small cohort of patients. (C) Kaplan–Meier curve with overall survival by tumor type.

Radiological responses according to RECIST 1.1 and OS are shown in the swimmer plot and OS also in Kaplan–Meier curves in Figure 1. The median OS for all patients was 8.4 months. The median OS for dose level 5 × 10^10^ VP was 4.4 months, dose level 1 × 10^11^ VP was 8.5 months and dose level 5 × 10^11^ VP was 14.5 months. Seven patients survived for at least 6 months after study inclusion, and four >12 months. OS per patient is listed in Supplementary Table S3, available at https://doi.org/10.1016/j.iotech.2026.101585.

**Figure 2:**
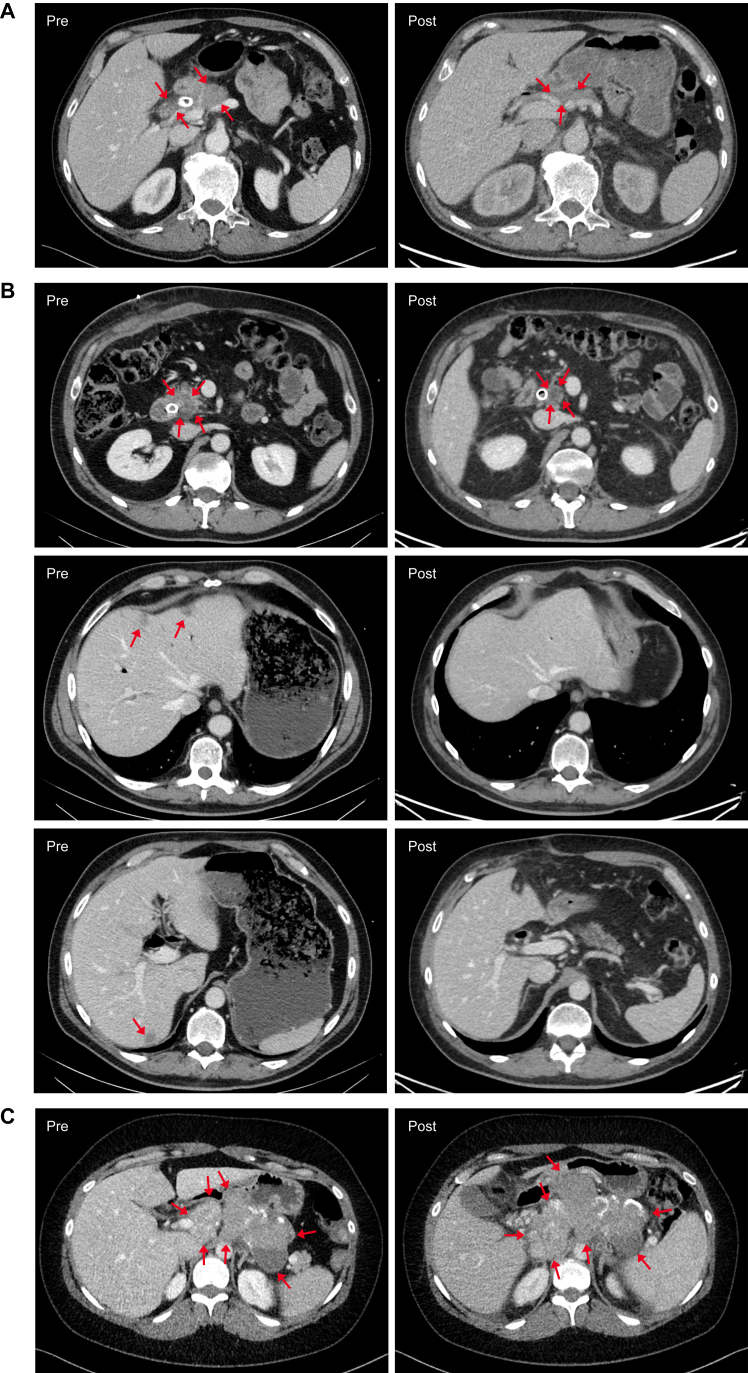
**CT images for three patients who experienced benefit from treatment.** (A) Patient PC-9 with locally advanced pancreatic cancer was treated with LOAd703 5 × 10^11^ VP combined with gemcitabine and nab-paclitaxel. The images show pancreatic tumor size at baseline (Pre) and 7 months after study inclusion with partial response according to RECIST1.1 (Post) with a decrease of 57%. (B) Patient PC-10 with liver-metastasized pancreatic cancer treated with LOAd703 5 × 10^11^ VP combined with gemcitabine and nab-paclitaxel. The top row of images shows pancreatic tumor size at baseline (Pre) and 6 months after study inclusion with partial response according to RECIST1.1 (Post) with a total decrease of 71%. The second and third row show images of liver metastases from the same time points. (C) Patient OC-5 with metastasized ovarian cancer treated with LOAd703 1 × 10^11^ VP, initially combined with gemcitabine. Gemcitabine was discontinued during study participation due to side-effects. The images show lymph node metastases at baseline (Pre) and stable disease 9 months later (Post) according to RECIST1.1. The lesions followed according to RECIST 1.1 had then increased in size by 14%. CT, computed tomography.

Best radiological response according to RECIST 1.1 was partial response (PR), achieved in two patients (PC-9 and PC-10) whose imaging is shown in Figure 2A and B. The best overall response per patient is listed in Supplementary Table S4, available at https://doi.org/10.1016/j.iotech.2026.101585. Both patients who experienced PR had pancreatic cancer and received LOAd703 at the highest dose level (5 × 10^11^ VP) in combination with gemcitabine and nab-paclitaxel as standard-of-care treatment in the first line. Detailed information on the number of LOAd703 and chemotherapy doses these patients received is provided in Supplementary Figure S1A, available at https://doi.org/10.1016/j.iotech.2026.101585. Patient PC-9 had locally advanced pancreatic cancer and continued with gemcitabine and nab-paclitaxel for 3 months after the final dose of LOAd703. This patient experienced PR 5 months after initiation of LOAd703, 2 months after the final LOAd703 dose when still receiving chemotherapy. The tumor mass was measured to decrease by 57% before it became difficult to distinguish from healthy pancreas and was reported as non-measurable at the final imaging. The patient remained treatment free for 17 months until gemcitabine was restarted due to disease progression. By then the tumor had increased in size locally as well as metastasized to the lungs. This patient reached an OS of >5 years (68 months). The other responder, PC-10, had pancreatic cancer with liver metastases and experienced PR 3.5 months after initiation of LOAd703, 1 week after the final LOAd703 injection, and was at the time still receiving chemotherapy. The injected lesion in the pancreas decreased by 39%, and the two non-injected liver metastases followed according to RECIST 1.1 became undetectable. Disease progression was evident 3.5 months after PR was achieved, and the patient continued chemotherapy per protocol until PD. No trend in response by lesion type was detected in this small patient material. Changes in tumor size of injected and non-injected lesions for the two patients who experienced PR are shown in Supplementary Figure S1B and C, available at https://doi.org/10.1016/j.iotech.2026.101585.

Another interesting case is patient PC-1 who had pancreatic cancer and was treated with LOAd703 at the lowest dose level combined with gemcitabine conditioning. During treatment, the tumor marker carbohydrate antigen 19-9 declined (1238 kE/l to nadir 507). Patient OC-5, suffering from ovarian cancer, reached an OS of 40 months. This patient had to stop conditioning gemcitabine chemotherapy 2 months after start of study treatment due to liver toxicity, and continued treatment with intratumoral injections of LOAd703 (1 × 10^11^ VP) alone. The tumor marker cancer antigen 125 declined during and after treatment, with the lowest value measured 2 months after the final dose of LOAd703 (238 kE/l to nadir 160 kE/l) and the patient experienced stable disease for 6 months after the final LOAd703 injection. During this time the patient received no other treatment. Imaging of this patient is shown in Figure 2C.

### Immune biomarkers

**Figure 3:**
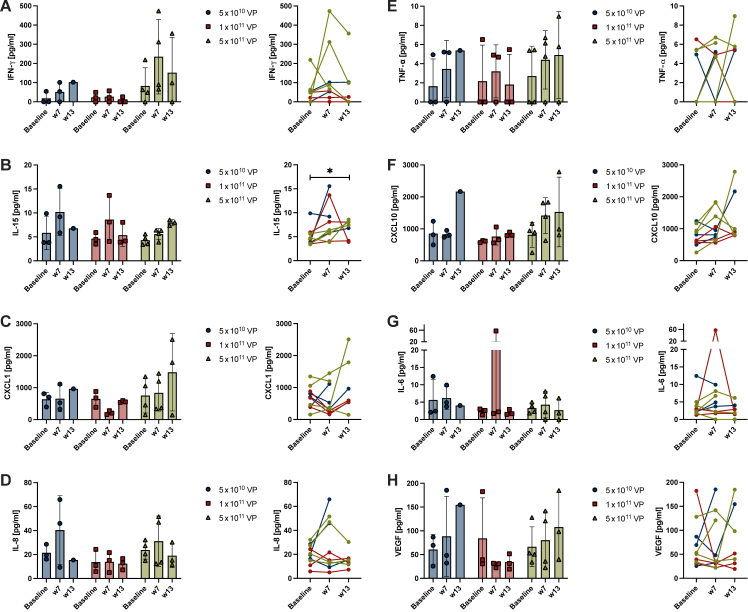
**Serum immune markers.** Serum sampled at baseline, week 7 and week 13 were analyzed by MesoScale technology for several immune markers (A-H). Results were sub-analyzed according to dose level and displayed in staple diagram. The change of values from baseline over time and across dose levels are shown to the side of each staple. Statistical calculations were made using two-way ANOVA (staple diagram; at each time point comparing dose levels) or one-way ANOVA (fold change; across dose levels comparing time points), both with Tukey’s multicomparison test. ANOVA, analysis of variance; IFN, interferon; IL, interleukin; TNF, tumor necrosis factor; VEGF, vascular endothelial growth factor; VP, viral particles; w, week.

Plasma concentrations of immune biomarkers were determined using MesoScale Diagnostics technology for individual patients with available samples. Immune biomarker levels were compared among the three dose levels (Figure 3). No statistically significant differences between the dose cohorts were detected. Nevertheless, at week 7 there was a trend of higher IFN-γ levels in the highest dose cohort of 5 × 10^11^ VP compared with the lower 1 × 10^11^ VP cohort (*P* = 0.0573). The two patients with PRs in the 5 × 10^11^ VP dose cohort were among patients with the highest IFN-γ levels. Of note, the high IL-6 value in the 1 × 10^11^ VP cohort reflects the patient who developed CRS (onset 2 weeks earlier).

## Discussion

As the immunotherapy field evolved with the introduction of checkpoint blockade antibodies, it became evident that mainly patients with pre-existing tumor-infiltrating lymphocytes (TILs) respond to treatment. Further, initially responding patients can develop secondary resistance.^1^ Hence, patients with low or few TILs at baseline or secondary resistance need other treatment strategies. TME gene engineering with mRNA or viral vectors is currently being evaluated as activating immunotherapies aiming to introduce *de novo* immune responses in patients with so-called ‘immune-cold’ TMEs.^2^ LOAd703 has two antitumor effector mechanisms. Firstly, it can cause direct cell death by oncolysis or TMZ-CD40L-mediated apoptosis. Secondly, LOAd703 can cause immune cell activation by expression of the transgenes in the TME and by immunogenicity of the viral backbone itself. Upon intratumoral injection, LOAd703 will infect both tumor cells and stromal cells, such as fibroblasts, endothelial cells, epithelial cells and myeloid cells, in the injected lesion. Stromal cells will not undergo oncolysis as they have an intact retinoblastoma pathway, but they may be killed by anti-adenovirus immune responses. A reduction of tumor stroma may lead to a better effect of the chemotherapeutic treatment. The infected stromal cells will also express TMZ-CD40L and 4-1BBL. *In vitro*, stellate cells from human pancreas infected with the LOAd703 virus were shown to reduce molecules that drive tumor progression including TGF-β, secreted phosphoprotein 1, galectin-3, hepatocyte growth factor and collagen type I while increasing chemokines involved to attract immune cells.^6^

Intratumoral treatment with the TME gene-engineering vector LOAd703 in combination with chemotherapy was feasible and well tolerated in patients with advanced cancer. The side-effects attributed to LOAd703 could be distinguished from chemotherapy based on previous knowledge. However, one limitation is that some AEs could potentially be attributed to both LOAd703 and chemotherapy. Nevertheless, there was no obvious overlap of AEs, but rather a distinct immune-related AE profile of LOAd703 that was manageable. The safety profile was similar at all three dose levels tested. Most AEs were transient and of low grade. The most common side-effects related to LOAd703 are all expected reactions to treatment with viral medicinal products and caused by an immediate immune system activation.^15^ A similar safety profile was observed in the LOKON001 study in pancreatic cancer patients treated with LOAd703, as well as previous phase I/II studies investigating an adenoviral vector encoding CD40L in multiple solid cancers.^14^^,^^16^^,^^17^ Furthermore, the phase III study (OPTIM) treating melanoma patients with the oncolytic herpes simplex virus talimogene laherparepvec (T-VEC) showed AEs resembling those in our study. T-VEC is a tumor-selective replication-competent virus which is modified to generate the expression of granulocyte–macrophage colony-stimulating factor in tumor cells and is now approved for treatment of metastatic melanoma.^18^ Although LOAd703 is injected intratumorally, some VP will leak out into the circulation, and symptoms such as pyrexia and chills evolving shortly after LOAd703 injections are likely due to antibody-mediated neutralization of the leaked VP. Leakage of VP into the circulation may be more prominent when injecting LOAd703 into liver metastases compared with other metastatic sites due to the higher vascularization. Nevertheless, no DLTs were reported, hence, the maximum tolerated dose was not reached.

CRS is a potential life-threatening complication of immunotherapy. One of the driving cytokines of CRS is IL-6 which is released upon immune cell activation.^19^ If CRS occurs secondary to LOAd703 treatment, it should be transient as LOAd703 leaking into the blood stream can rapidly be cleared by the neutralizing antibodies that drive the CRS reaction. CRS can be managed by treatment with immunosuppressive medication such as corticosteroids and/or antibodies to cytokines or their receptors such as the IL-6 receptor inhibitor tocilizumab.^19^ One of the patients in this study developed CRS grade 2. The CRS symptoms subsided quickly after administration of tocilizumab. This patient subsequently received premedication with corticosteroids, which allowed all planned LOAd703 injections to be administered without complications. The impact of anti-inflammatory treatment on the efficacy of immunotherapy has shown contradictory results.^19^ The patient who developed CRS in our study showed no effect from treatment, but this patient was also heavily pretreated at inclusion. The dose-escalation phase of this study supported a handling plan for CRS.

A dose-limiting factor for systemic treatment with CD40- and 4-1BB-targeting therapy has been hepatotoxicity with increasing levels of liver transaminases, and two treatment-related deaths in a study with a 4-1BB agonistic antibody.^16^^,^^17^^,^^20^, ^21^, ^22^, ^23^ In our cohort of 10 patients, no increase in liver transaminases attributed to LOAd703 was reported, indicating that the local expression of TMZ-CD40L and 4-1BBL, rather than intravenous administration resulting in high systemic exposure, is a safer option. The overall mild toxicity profile in this heterogeneous patient group implies that LOAd703 can be safely tested in a broad spectrum of cancer indications using intratumoral delivery.

Our study had a similar safety profile as the other reported study of LOAd703 in pancreatic cancer patients.^14^ One aspect which differed between these two studies was the timing of the first LOAd703 dose correlated to chemotherapy. In our study, LOAd703 was administered directly on day 1 in chemotherapy cycle 1. In the other reported study, LOAd703 was administered first on day 15 of the first chemotherapy cycle. Administering a few doses of chemotherapy before introducing LOAd703 could potentially reduce some of the initial antiviral immune response compared with if LOAd703 is introduced directly. One event of CRS was reported in our study, as opposed to none in the previously reported study in pancreatic cancer patients, which can be a sign of increased antiviral immune response when giving LOAd703 directly on day 1 of chemotherapy cycle 1. However, the timing of LOAd703 introduction does not seem to substantially impact safety. Our study further demonstrates that LOAd703 in addition to being safe in pancreatic cancer patients treated with standard-of-care chemotherapy can be safely administered to both colorectal and ovarian cancer patients, as well as more advanced pancreatic cancer patients not suitable for the combination therapy gemcitabine and nab-paclitaxel.

The clinical results are descriptive only and should be interpreted with caution in this small heterogeneous group of patients. The treatment benefit seemed to improve with increasing doses of LOAd703. Nevertheless, a limitation to this interpretation is the different chemotherapy regimens used in combination with LOAd703 in the two lower versus the highest dose cohort. Two patients with pancreatic cancer experienced radiological PRs. These patients received the same treatment regimen, LOAd703 5 × 10^11^ VP combined with gemcitabine and nab-paclitaxel. It is not possible to determine whether the tumor response was attributed to chemotherapy alone, which has a 23% expected response rate on its own in treatment-naive patients with metastatic pancreatic cancer.^24^ Nevertheless, the sustained response seen in one of these patients is not expected from chemotherapy alone. The response was ongoing for 17 months and the patient survived for >5 years from study enrollment. It is likely that the LOAd703 contributed to the long-lasting treatment benefit.

The patient with ovarian cancer who experienced long-lasting stable disease had already been treated with several lines of therapy and therefore received gemcitabine as an immune-conditioning chemotherapy with LOAd703. This patient stopped gemcitabine treatment due to liver toxicity and received the majority of LOAd703 injections without conditioning and with no further liver toxicity. Interestingly, her tumor marker decreased during treatment with LOAd703 alone and she had stable disease for several months which supports the conclusion that this patient experienced benefit from LOAd703 monotreatment.

Immune biomarker analyses demonstrated high plasma levels of serum IFN-γ in patients receiving the highest dose of LOAd703. Notably, the two patients who experienced PR were in the highest-dose group and developed among the highest increases of IFN-γ. However, statistically significant differences at a 95% confidence interval among dose cohorts were not noted in this small sample size, but will be interesting to follow in larger cohorts in phase II. That two different chemotherapy regimens were used in this study could potentially affect IFN-γ levels, as chemotherapy may reduce immunosuppressive immune cell populations. However, the elevation in IFN-γ levels is in line with previous *in vitro* models with LOAd703 alone, which have shown that LOAd703-treated DCs have an increased IFN-γ secretion that is important in the Th1 immune response.^6^^,^^8^ Clinical phase I/II studies with an adenoviral vector encoding CD40L have also shown a trend of increased IFN-γ or IFN-γ-induced cytokines in tumor biopsies after administration.^16^^,^^25^ Based on the data presented here, and considering previous *in vitro* data, LOAd703 exhibits potential to stimulate clinically meaningful immune activation. Increase in IFN-γ was not coupled to the safety profile of LOAd703. However, further analyses are currently conducted in order to map the local and systemic immune reactions induced by LOAd703.

In summary, this study demonstrates that intratumoral injections of LOAd703 in combination with standard-of-care or conditioning chemotherapy were well tolerated at all dose levels tested. The maximum tolerated dose was not reached. An acceptable toxicity profile in combination with signs of clinical efficacy in patients with advanced pancreatic and ovarian cancer warrants further studies with LOAd703.
