# Supplementary material for: Safety of intratumoral immunostimulatory LOAd703 gene therapy combined with chemotherapy in patients with advanced cancer
Source: Immunooncol Technol. 2026 Feb 6;29:101585. doi: 10.1016/j.iotech.2026.101585 (PMC12969339; doi:10.1016/j.iotech.2026.101585)
Supplement: Supplementary Figures and Tables [file mmc1.pdf]

# Supplementary Material

## Safety of intratumoral immunostimulatory LOAd703 gene therapy combined with chemotherapy in patients with advanced cancer

Amanda Hahn*, Sandra Irenaeus*, Linda C. Sandin, Jessica Wenthe, Emma Eriksson, Justyna Leja Jarblad, Aglaia Schiza, Hanna Dahlstrand, Ulla Olsson-Strömberg, Johan Krause, Anders Sundin, Angelica Loskog* and Gustav J. Ullenhag*

*Contributed equally

Supplementary Table S1: All adverse events

Supplementary Table S2: Adverse events related to LOAd703 per patient and injection

Supplementary Table S3: Overall survival by patient

Supplementary Table S4: Best overall response

Supplementary Figure S1. Treatment details and tumor size change for the two patients who experienced partial response

## Supplementary Table S1: All adverse events

| Type of AE | Total, N (%) of patients[a] with | | | | | Total Number of AEs |
|---|---|---|---|---|---|---|
| | AE Grade 1-2 | AE Grade 3-4 | SAE Grade 1-2 | SAE Grade 3-4 | AE Any grade | |
| ***Dose level: 5x10$^{10}$ VP (n=3)*** | | | | | | |
| Chills | 3 (100%) | 0 (0%) | 0 (0%) | 0 (0%) | 3 (100%) | 7 |
| Pyrexia | 3 (100%) | 0 (0%) | 1 (33%) | 0 (0%) | 3 (100%) | 12 |
| Back pain | 1 (33%) | 1 (33%) | 0 (0%) | 1 (33%) | 2 (67%) | 3 |
| Dyspnoea | 1 (33%) | 1 (33%) | 0 (0%) | 0 (0%) | 2 (67%) | 2 |
| Fatigue | 2 (67%) | 1 (33%) | 1 (33%) | 0 (0%) | 2 (67%) | 6 |
| Hypertension | 2 (67%) | 1 (33%) | 0 (0%) | 0 (0%) | 2 (67%) | 4 |
| Myalgia | 2 (67%) | 0 (0%) | 0 (0%) | 0 (0%) | 2 (67%) | 3 |
| Nausea | 2 (67%) | 0 (0%) | 0 (0%) | 0 (0%) | 2 (67%) | 2 |
| Oedema peripheral | 2 (67%) | 0 (0%) | 0 (0%) | 0 (0%) | 2 (67%) | 2 |
| Vomiting | 2 (67%) | 0 (0%) | 0 (0%) | 0 (0%) | 2 (67%) | 7 |
| Abdominal pain | 0 (0%) | 1 (33%) | 0 (0%) | 1 (33%) | 1 (33%) | 1 |
| Anaemia | 1 (33%) | 0 (0%) | 0 (0%) | 0 (0%) | 1 (33%) | 1 |
| Arthralgia | 1 (33%) | 0 (0%) | 0 (0%) | 0 (0%) | 1 (33%) | 1 |
| Constipation | 1 (33%) | 0 (0%) | 0 (0%) | 0 (0%) | 1 (33%) | 1 |
| Decreased appetite | 1 (33%) | 0 (0%) | 0 (0%) | 0 (0%) | 1 (33%) | 1 |
| General physical health deterioration | 0 (0%) | 1 (33%) | 0 (0%) | 1 (33%) | 1 (33%) | 2 |
| Headache | 1 (33%) | 0 (0%) | 0 (0%) | 0 (0%) | 1 (33%) | 1 |
| Lung infiltration | 1 (33%) | 0 (0%) | 0 (0%) | 0 (0%) | 1 (33%) | 1 |
| Malaise | 1 (33%) | 0 (0%) | 0 (0%) | 0 (0%) | 1 (33%) | 1 |
| Palpitations | 1 (33%) | 0 (0%) | 0 (0%) | 0 (0%) | 1 (33%) | 1 |
| Proctalgia | 1 (33%) | 0 (0%) | 0 (0%) | 0 (0%) | 1 (33%) | 1 |
| Stomatitis | 1 (33%) | 0 (0%) | 0 (0%) | 0 (0%) | 1 (33%) | 1 |
| ***Dose level: 1x10$^{11}$ VP (n=3)*** | | | | | | |
| Fatigue | 2 (67%) | 0 (0%) | 0 (0%) | 0 (0%) | 2 (67%) | 3 |
| Headache | 2 (67%) | 0 (0%) | 0 (0%) | 0 (0%) | 2 (67%) | 7 |
| Nausea | 2 (67%) | 0 (0%) | 0 (0%) | 0 (0%) | 2 (67%) | 3 |
| Oedema peripheral | 2 (67%) | 0 (0%) | 1 (33%) | 0 (0%) | 2 (67%) | 2 |
| Pyrexia | 2 (67%) | 0 (0%) | 0 (0%) | 0 (0%) | 2 (67%) | 2 |
| Abdominal pain | 1 (33%) | 0 (0%) | 0 (0%) | 0 (0%) | 1 (33%) | 1 |
| C-reactive protein increased | 1 (33%) | 0 (0%) | 0 (0%) | 0 (0%) | 1 (33%) | 1 |
| Cytokine release syndrome | 1 (33%) | 0 (0%) | 1 (33%) | 0 (0%) | 1 (33%) | 1 |
| Decreased appetite | 1 (33%) | 0 (0%) | 0 (0%) | 0 (0%) | 1 (33%) | 1 |
| Discomfort | 1 (33%) | 0 (0%) | 0 (0%) | 0 (0%) | 1 (33%) | 2 |
| Electrocardiogram abnormal | 1 (33%) | 0 (0%) | 0 (0%) | 0 (0%) | 1 (33%) | 1 |
| Gastroenteritis | 1 (33%) | 0 (0%) | 0 (0%) | 0 (0%) | 1 (33%) | 1 |
| Groin pain | 0 (0%) | 1 (33%) | 0 (0%) | 0 (0%) | 1 (33%) | 1 |
| Haematuria | 0 (0%) | 1 (33%) | 0 (0%) | 1 (33%) | 1 (33%) | 1 |
| Hepatic enzyme increased | 1 (33%) | 1 (33%) | 0 (0%) | 1 (33%) | 1 (33%) | 2 |
| Hypotension | 1 (33%) | 0 (0%) | 0 (0%) | 0 (0%) | 1 (33%) | 1 |
| Injection site oedema | 1 (33%) | 0 (0%) | 0 (0%) | 0 (0%) | 1 (33%) | 1 |
| Leukopenia | 0 (0%) | 1 (33%) | 0 (0%) | 0 (0%) | 1 (33%) | 1 |

| | | | | | | |
|---|---|---|---|---|---|---|
| Lung infiltration | 1 (33%) | 0 (0%) | 0 (0%) | 0 (0%) | 1 (33%) | 1 |
| Myalgia | 1 (33%) | 0 (0%) | 0 (0%) | 0 (0%) | 1 (33%) | 1 |
| Nasopharyngitis | 1 (33%) | 0 (0%) | 0 (0%) | 0 (0%) | 1 (33%) | 1 |
| Neutropenia | 0 (0%) | 1 (33%) | 0 (0%) | 1 (33%) | 1 (33%) | 1 |
| Oropharyngeal pain | 1 (33%) | 0 (0%) | 0 (0%) | 0 (0%) | 1 (33%) | 1 |
| Pain in extremity | 0 (0%) | 1 (33%) | 0 (0%) | 0 (0%) | 1 (33%) | 1 |
| Sinus tachycardia | 1 (33%) | 0 (0%) | 0 (0%) | 0 (0%) | 1 (33%) | 1 |
| Urinary tract infection | 1 (33%) | 0 (0%) | 0 (0%) | 0 (0%) | 1 (33%) | 1 |
| Vomiting | 1 (33%) | 0 (0%) | 0 (0%) | 0 (0%) | 1 (33%) | 1 |
| ***Dose level: $5x10^{11}$ VP (n=4)*** | | | | | | |
| Pyrexia | 4 (100%) | 0 (0%) | 1 (25%) | 0 (0%) | 4 (100%) | 18 |
| C-reactive protein increased | 3 (75%) | 0 (0%) | 0 (0%) | 0 (0%) | 3 (75%) | 3 |
| Fatigue | 3 (75%) | 0 (0%) | 0 (0%) | 0 (0%) | 3 (75%) | 4 |
| Nausea | 3 (75%) | 0 (0%) | 0 (0%) | 0 (0%) | 3 (75%) | 3 |
| Anaemia | 1 (25%) | 1 (25%) | 0 (0%) | 0 (0%) | 2 (50%) | 4 |
| Headache | 2 (50%) | 0 (0%) | 0 (0%) | 0 (0%) | 2 (50%) | 3 |
| Lung infiltration | 2 (50%) | 0 (0%) | 0 (0%) | 0 (0%) | 2 (50%) | 2 |
| Oedema peripheral | 2 (50%) | 0 (0%) | 0 (0%) | 0 (0%) | 2 (50%) | 2 |
| Abdominal pain | 0 (0%) | 1 (25%) | 0 (0%) | 1 (25%) | 1 (25%) | 1 |
| Abdominal pain upper | 0 (0%) | 1 (25%) | 0 (0%) | 0 (0%) | 1 (25%) | 1 |
| Back pain | 1 (25%) | 0 (0%) | 0 (0%) | 0 (0%) | 1 (25%) | 1 |
| Blood creatinine increased | 1 (25%) | 1 (25%) | 0 (0%) | 1 (25%) | 1 (25%) | 2 |
| Chills | 1 (25%) | 0 (0%) | 0 (0%) | 0 (0%) | 1 (25%) | 3 |
| Constipation | 1 (25%) | 0 (0%) | 1 (25%) | 0 (0%) | 1 (25%) | 1 |
| Cough | 1 (25%) | 0 (0%) | 0 (0%) | 0 (0%) | 1 (25%) | 2 |
| Cystitis | 0 (0%) | 1 (25%) | 0 (0%) | 0 (0%) | 1 (25%) | 1 |
| Decreased appetite | 1 (25%) | 0 (0%) | 0 (0%) | 0 (0%) | 1 (25%) | 1 |
| Diarrhoea | 1 (25%) | 0 (0%) | 0 (0%) | 0 (0%) | 1 (25%) | 1 |
| Erysipelas | 1 (25%) | 0 (0%) | 0 (0%) | 0 (0%) | 1 (25%) | 1 |
| Face oedema | 1 (25%) | 0 (0%) | 0 (0%) | 0 (0%) | 1 (25%) | 1 |
| General physical health deterioration | 1 (25%) | 0 (0%) | 0 (0%) | 0 (0%) | 1 (25%) | 1 |
| Generalised oedema | 1 (25%) | 0 (0%) | 0 (0%) | 0 (0%) | 1 (25%) | 1 |
| Groin infection | 1 (25%) | 0 (0%) | 0 (0%) | 0 (0%) | 1 (25%) | 1 |
| Hyperkalaemia | 0 (0%) | 1 (25%) | 0 (0%) | 1 (25%) | 1 (25%) | 1 |
| Hypertension | 0 (0%) | 1 (25%) | 0 (0%) | 0 (0%) | 1 (25%) | 1 |
| Hypokalaemia | 1 (25%) | 0 (0%) | 0 (0%) | 0 (0%) | 1 (25%) | 1 |
| Hypotension | 1 (25%) | 0 (0%) | 0 (0%) | 0 (0%) | 1 (25%) | 2 |
| Ileus | 0 (0%) | 1 (25%) | 0 (0%) | 1 (25%) | 1 (25%) | 1 |
| Incisional hernia | 1 (25%) | 0 (0%) | 0 (0%) | 0 (0%) | 1 (25%) | 1 |
| Infection | 0 (0%) | 1 (25%) | 0 (0%) | 1 (25%) | 1 (25%) | 1 |
| Influenza like illness | 1 (25%) | 0 (0%) | 0 (0%) | 0 (0%) | 1 (25%) | 1 |
| Injection site inflammation | 1 (25%) | 0 (0%) | 0 (0%) | 0 (0%) | 1 (25%) | 1 |
| Localised oedema | 1 (25%) | 0 (0%) | 0 (0%) | 0 (0%) | 1 (25%) | 1 |
| Myalgia | 1 (25%) | 0 (0%) | 0 (0%) | 0 (0%) | 1 (25%) | 1 |
| Neuropathy peripheral | 1 (25%) | 0 (0%) | 0 (0%) | 0 (0%) | 1 (25%) | 1 |
| Periorbital oedema | 1 (25%) | 0 (0%) | 0 (0%) | 0 (0%) | 1 (25%) | 1 |

| | | | | | | |
|---|---|---|---|---|---|---|
| Pulmonary embolism | 0 (0%) | 1 (25%) | 0 (0%) | 0 (0%) | 1 (25%) | 1 |
| Renal failure | 0 (0%) | 1 (25%) | 0 (0%) | 1 (25%) | 1 (25%) | 2 |
| Swelling | 1 (25%) | 0 (0%) | 0 (0%) | 0 (0%) | 1 (25%) | 1 |
| Thrombocytosis | 1 (25%) | 0 (0%) | 0 (0%) | 0 (0%) | 1 (25%) | 1 |
| Tumour pain | 1 (25%) | 0 (0%) | 0 (0%) | 0 (0%) | 1 (25%) | 1 |
| Urinary retention | 1 (25%) | 0 (0%) | 0 (0%) | 0 (0%) | 1 (25%) | 1 |
| Urinary tract infection | 1 (25%) | 0 (0%) | 0 (0%) | 0 (0%) | 1 (25%) | 1 |
| Vomiting | 1 (25%) | 0 (0%) | 0 (0%) | 0 (0%) | 1 (25%) | 1 |
| Weight decreased | 1 (25%) | 0 (0%) | 0 (0%) | 0 (0%) | 1 (25%) | 1 |
| Weight increased | 1 (25%) | 0 (0%) | 0 (0%) | 0 (0%) | 1 (25%) | 1 |

AEs were graded according to CTCAE Version 4.03. Data are n (%) unless otherwise specified. Worst grade is included in the table. Coded according to Medical Dictionary for Regulatory Activities (MedDRA v21.0).
Abbreviations: AE, adverse event; SAE, serious adverse event.
[a]Safety-evaluable patients. Data cut-off: November 30, 2023

## Supplementary Table S2: Adverse events related to LOAd703 per patient and injection

| Patient ID | Injection 1 | Injection 2 | Injection 3 | Injection 4 | Injection 5 | Injection 6 | Injection 7 | Injection 8 |
|---|---|---|---|---|---|---|---|---|
| **PC-1** | Chills G1<br>Pyrexia G1 | Chills G2<br>Pyrexia G2<br>Vomiting G2<br>Malaise G2<br>Myalgia G2 | Pyrexia G1 | Pyrexia G1<br>Fatigue G1 | Chills G2<br>Pyrexia G2<br>Vomiting G1 | Chills G2<br>Pyrexia G1 | Chills G2<br>Pyrexia G2<br>Vomiting G1 | |
| **CRC-2** | Myalgia G1<br>Chills G1<br>Pyrexia G2 | Myalgia G1 | Fatigue G2 | | | | | |
| **CRC-3** | Chills G2<br>Pyrexia G2 | | | Pyrexia G1 | | | | |
| **CRC-4** | | Fatigue G1 | Cytokine release syndrome G2<br>Nausea G1<br>Vomiting G1 | Pyrexia G1<br>Hypotension G1<br>Sinus tachycardia G1 | | | | |
| **OC-5** | Headache G1<br>Discomfort G1 | C-reactive protein increased G2<br>Headache G1 | Headache G1 | | Discomfort G1 | Headache G1 | Headache G1 | Headache G1 |
| **OC-7** | Hypotension G1<br>Pyrexia G1<br>Back pain G1 | Chills G2<br>Pyrexia G2<br>Vomiting G1<br>Hypotension G2 | Chills G2 | | | | | |
| **PC-8** | Pyrexia G1 | Headache G1 | | | | | | |
| **PC-9** | | | Pyrexia G1 | Pyrexia G2 | Pyrexia G1 | | | |
| **PC-10** | | | | | | | Fatigue G2 | Pyrexia G1<br>Headache G1<br>Myalgia G1 |

AEs were graded according to CTCAE Version 4.03 and coded according to Medical Dictionary for Regulatory Activities (MedDRA v21.0). AEs reported are those which developed the same day or the day after LOAd703 injection, i.e. shortly after administration, and reported as "possibly related," "probably related", or "definitely related" to LOAd703. AEs are reported per patient and number of injection. CRC-6 only had one AE related to LOAd703 which did not develop within the first two days, this patient is therefore not included in the table.
Abbreviations: G, Grade
Data cut-off: November 30, 2023

## Supplementary Table S3: Overall survival by patient

| Patient ID | Tumor type | Overall survival (months) |
|---|---|---|
| ***Dose level: 5x10$^{10}$ VP*** | | |
| **PC-1** | Pancreatic cancer | 12.9 |
| **CRC-2** | Colorectal carcinoma | 4.4 |
| **CRC-3** | Colorectal carcinoma | 3.0 |
| ***Dose level: 1x10$^{11}$ VP*** | | |
| **CRC-4** | Colorectal carcinoma | 8.4 |
| **OC-5** | Ovarian cancer | 40.6 |
| **CRC-6** | Colorectal carcinoma | 8.5 |
| ***Dose level: 5x10$^{11}$ VP*** | | |
| **OC-7** | Ovarian cancer | 7.6 |
| **PC-8** | Pancreatic cancer | 2.9 |
| **PC-9** | Pancreatic cancer | 67.9 |
| **PC-10** | Pancreatic cancer | 21.5 |

Data cut-off: May 27, 2025.

## Supplementary Table S4: Best overall response

| Patient ID | Target lesions | Nontarget lesions | New lesions | Overall response |
|---|---|---|---|---|
| ***Dose level: 5x$10^{10}$ VP*** | | | | |
| **PC-1** | SD | NA | No | Stable disease |
| **CRC-2** | PD | Present | Yes | Progressive disease |
| **CRC-3** | PD | Present | Yes | Progressive disease |
| ***Dose level: 1x$10^{11}$ VP*** | | | | |
| **CRC-4** | SD | Present | No | Stable disease |
| **OC-5** | SD | Present | No | Stable disease |
| **CRC-6** | PD | Present | No | Progressive disease |
| ***Dose level: 5x$10^{11}$ VP*** | | | | |
| **OC-7** | SD | Present | No | Stable disease |
| **PC-8** | PD | Present | Yes | Progressive disease |
| **PC-9** | PR | NA | No | Partial response |
| **PC-10** | PR | Present | No | Partial response |

Anti-tumoral effects were evaluated according to Response Evaluation Criteria in Solid Tumors (RECIST) version 1.1. Best overall response obtained during the study is reported per patient. Each patient was followed until disease progression or maximum 40 weeks.
Abbreviations: SD, Stable disease; PD, Progressive disease; PR, Partial response

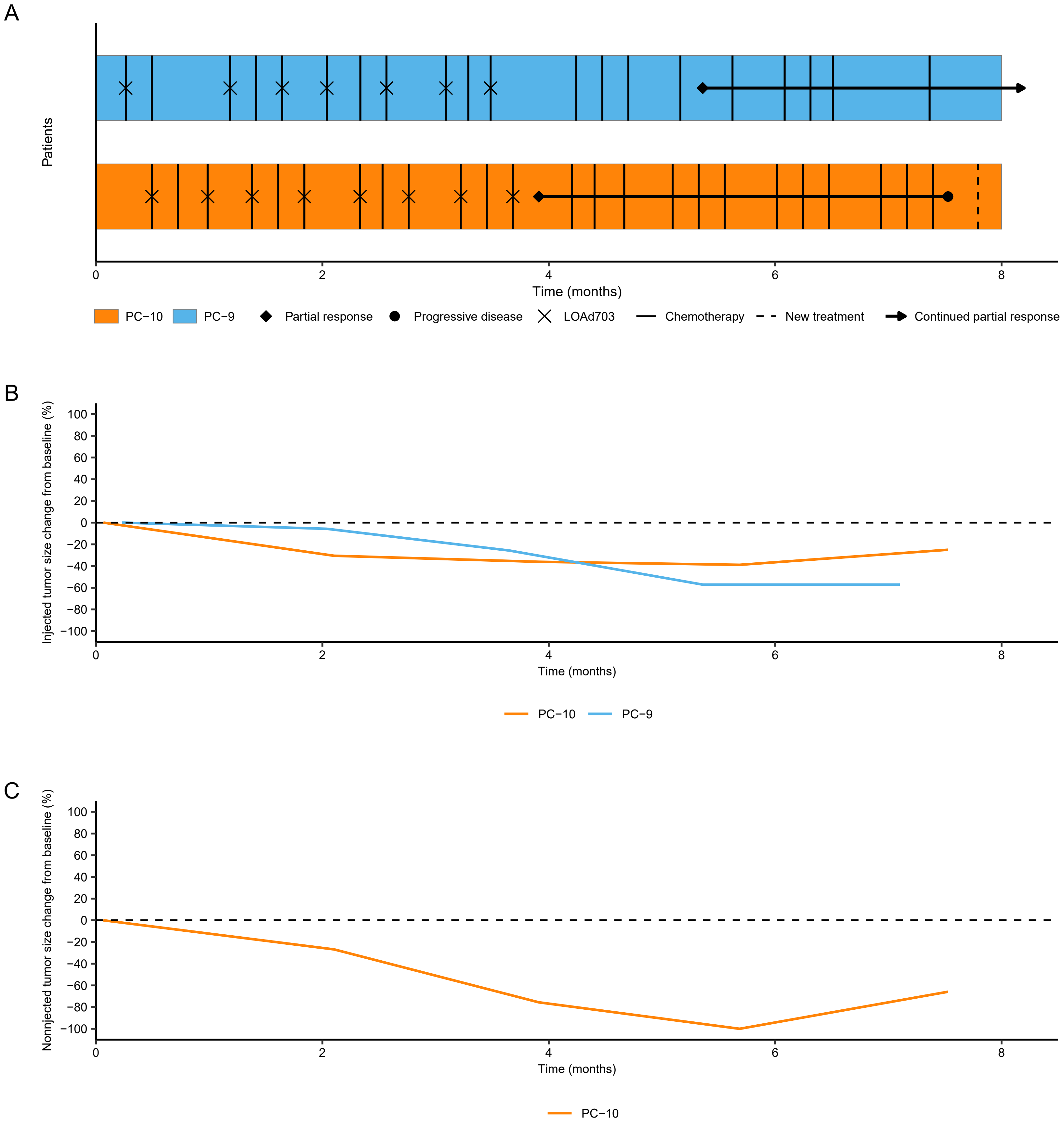


Supplementary Figure S1. **Treatment details and tumor size change for the two patients who experienced partial response.** Both, PC-9 and PC-10, had pancreatic cancer and received LOAd703 and gemcitabine plus nab-paclitaxel as first line treatment. A) Swimmer plot which shows the time points after study inclusion when PC-9 and PC-10 received LOAd703 and chemotherapy, in addition to the periods they experienced partial response. Patient PC-9 had locally advanced disease, experienced partial response five months after the first dose of LOAd703 and did not progress during the nine-month follow-up. At the final assessment, the tumor was non-measurable and not part of the Figure. Patient PC-10 had pancreatic cancer with metastases to the liver. This patient experienced partial response about three and a half months after the first LOAd703 injection and progressed after seven months. B) shows tumor size changes over time for lesions injected with LOAd703 in the two patients. Both received injections in the primary tumor in the pancreas. C) shows tumor size changes for the non-injected lesions followed according to RECIST 1.1 in patient PC-10.
